# Quantitative analysis of the eyelid curvature in patients with blepharoptosis

**DOI:** 10.1186/s12880-024-01280-x

**Published:** 2024-04-26

**Authors:** Elias Khalili Pour, Tahereh Mahmoudi, Hooman Ahmadzadeh, Seyed Mohsen Rafizadeh, Hamid Riazi-Esfahani

**Affiliations:** 1 Retina Service, Farabi Eye Hospital, Tehran University of Medical Sciences, Tehran, Iran; 2 Department of Medical Physics and Biomedical Engineering, School of Medicine, Shiraz University of Medical Sciences, Shiraz, Iran; 3 Eye Research Center, Farabi Eye Hospital, Tehran University of Medical Sciences, Tehran, Iran; 4 Orbital and Oculoplastics Service, Farabi Eye Hospital, Tehran University of Medical Sciences, Tehran, Iran

**Keywords:** Eyelid, Eyelid contour, Ptosis, Eyelid curvature

## Abstract

**Background:**

The aim of this study was to evaluate the ability of two novel eyelid curvature measurements to distinguish between normal eyes and different severities of blepharoptosis.

**Methods:**

A comparative cross-sectional analysis of upper eyelid curvature was performed for different severities of patients with unilateral blepharoptosis (congenital and aponeurotic) and normal controls. Mean upper lid contour index (ULCI) and area circularity index (ACI) were calculated for each group by dividing the intercanthal distance by upper eyelid margin length (ULCI) and dividing the interpalpebral area by the area of a circle enclosing the eye (ACI). The ratio of each index for the study and fellow normal eye of each patient was also calculated and compared between groups.

**Results:**

A total of 106 eyes including 30 eyes in the control group and 25, 27, and 24 eyes in the mild, moderate, and severe ptosis groups were enrolled in the study. ULCI and ACI showed a statistically significant difference between the groups (*p* < 0.001, *p* < 0.001). The inter-eye ratio (ULCI-ratio and ACI-ratio) of indices was also significantly different between groups (*p* = 0.002, *p* < 0.001). Pairwise comparisons revealed that ACI and ACI-ratio were significantly different between all pairs of study groups.

**Conclusion:**

The results of our study showed that ACI based on area measurements may distinguish blepharoptosis patients from normal controls and from each other. Including the data from the fellow normal eyes in the form of ratio indices may improve the differentiating power. These results can be useful in designing the optimal eyelid curvature measurements.

## Background

The eyelid is a protective barrier critical for maintaining a healthy ocular surface [1]. Changes in eyelid curvature and anatomy can have adverse cosmetic effects and disturb its protective function [2]. Various conditions affect the eyelid's curvature. Among these are blepharoptosis, Graves' ophthalmopathy, and eyelid tumors [3].

Blepharoptosis, a common indication for upper eyelid surgery, may have a myogenic, neurogenic, traumatic, or mechanical cause. The most prevalent acquired form, involutional blepharoptosis, is caused by age-related stretching or dehiscence of the levator aponeurosis from the anterior tarsal surface, resulting in upper eyelid drooping [4].

The margin reflex distance 1 (MRD1) is the most common method for determining eyelid position. It is the distance between the pupillary light reflex and the upper eyelid margin [5]. Despite their reliability, MRD measurements can be time-consuming and operator-dependent [6, 7]. Multiple techniques have been utilized to extract MRD1 values from digital images [8, 9]. However, MRD only describes the eyelid's central point and provides little information regarding the actual eyelid's curvature. Consequently, it cannot quantify specific contour anomalies like notches, peaks, and lateral flares. Such information is necessary for a comprehensive evaluation of the eyelid morphology, the diagnosis of abnormalities, surgical planning, and the evaluation of changes in the eyelid curvature after surgery.

Several studies have reported novel digital imaging analysis methods to address these limitations [10–15]. Milbratz et al. developed multiple radial mid pupil lid distances (MPLD) that assess the length of pupil center to eyelid margin at 15° intervals ranging from 0 to 180° [16]. Danesh et al. proposed multiple vertical lines parallel to MRD1 that measure eyelid height at multiple points medial and lateral to MRD1 [17]. Bezier curves, widely used in computer graphics, define a curve by a mathematical formula and have been suggested to analyze eyelid curvature [18–20]. However, a universally accepted method hasn’t yet emerged.

In the current study, we present new indices describing the eyelid contour and evaluate their effectiveness in discriminating eyelids with blepharoptosis from a control group.

## Methods

This study was conducted in Farabi Eye Hospital in Tehran, Iran. The Tehran University of Medical Sciences institutional review board and ethics committee approved the study, and the tenets of the Declaration of Helsinki were followed throughout the study. Written informed consent was acquired from all patients. For participants under 18, the informed consent was obtained from a parent or legal guardian.

### Patient selection

A comparative cross-sectional analysis of the upper eyelid contour was conducted on Iranian patients with blepharoptosis and healthy controls. Patients over the age of 3 with upper eyelid ptosis were retrospectively included in the study. Patients with any history of previous eyelid surgery, eyebrow and orbital disease, strabismus, any condition that might influence upper eyelid contour other than blepharoptosis such as thyroid orbitopathy and facial palsy, and a history of botulinum injection in the last six months were excluded from the study. Only patients with unilateral blepharoptosis were included in the study. Type of blepharoptosis for included patients were congenital and aponeurotic. As the control group, patients with no ocular abnormalities were included. The study eye for the control group was selected at random.

### Image analysis

Uncropped full-face photographs of patients were classified by consensus as mild, moderate, or severe ptosis by three ophthalmologists (S.M.R, H.R.E, and E.K.P). Using a camera system (Nikon D7500 Digital Camera With 18-140mm VR AF-S DX Lens) held at the pupil level between the patients' eyes at a distance of 25 cm, full-face images were taken of each patient while seated. All images were taken in the primary position of gaze. In order to facilitate the software algorithm's determination of the millimeters per pixel scale of the image plane, a measuring ruler in millimeters is positioned adjacent to the image capture location. To measure MRD1, the images were magnified by a computer. The ophthalmologists utilized ImageJ (National Institutes of Health, Bethesda, MD, USA) to draw a vertical line across the upper eyelid edge and pupillary light reflex. The measurement was performed in 0.1 mm intervals, and the mean value was used to compare the groups.

**Fig. 1:**
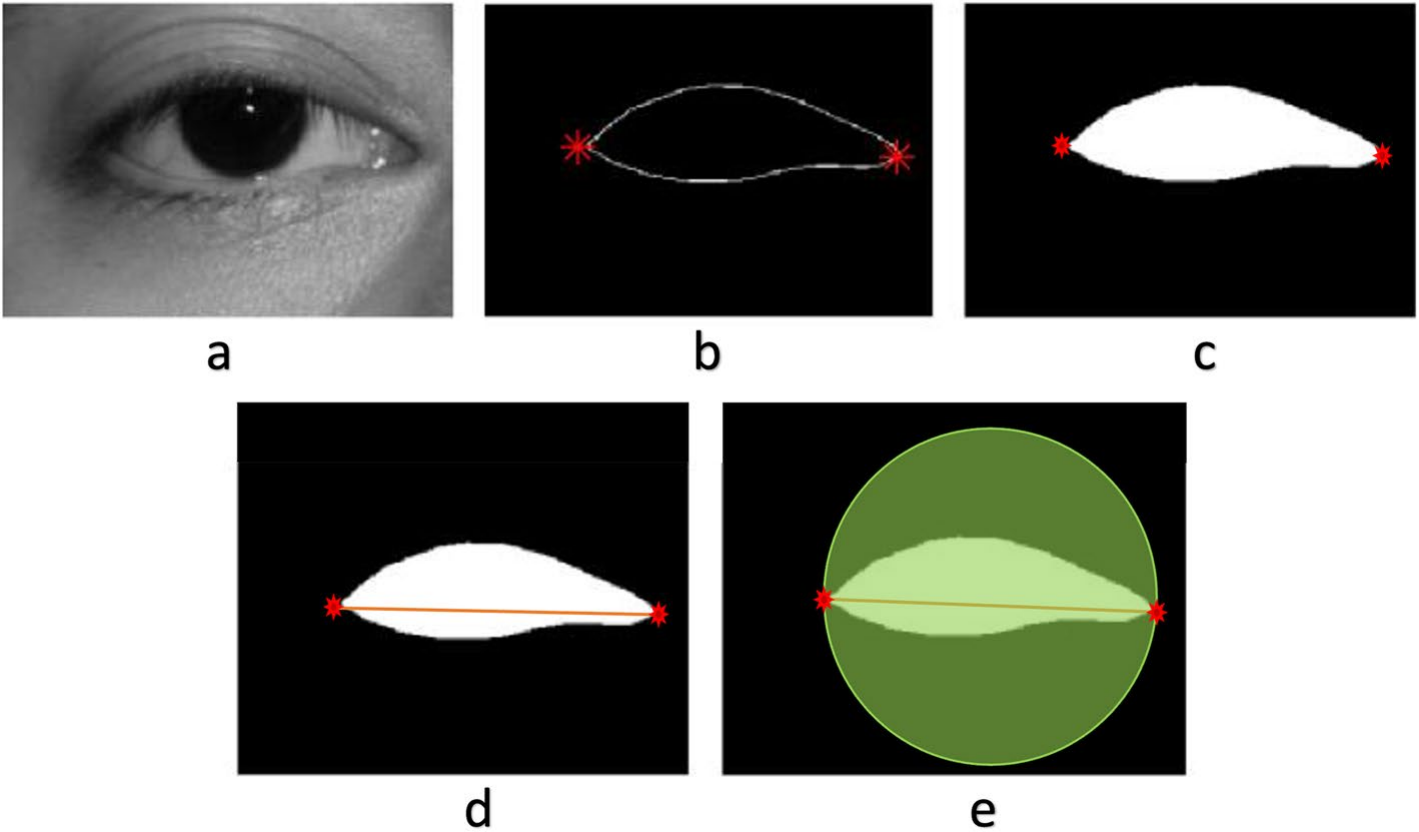
Example of preprocessing a digital photograph for deriving measured indices. The images were converted to grayscale (**a**). On the image, the operator has marked the medial and lateral canthi (**b**). To build a palpebral fissure region of interest (ROI) with an interactive polygonal drawing approach, the operator would first click on the medial or lateral canthus, then drag the mouse along the eyelid contour, and then click on the opposite canthus (**c**). The image was then binarized, and a circle was formed around the eye, with the connecting line between the medial and lateral canthi serving as the diameter (**d**, **e**)

The image processing techniques in this study for the eyelid curvature analysis were implemented using MATLAB software R2019 (MathWorks, Inc., Natick, MA). The images were converted to grayscale and enhanced using homomorphic filter and normalization methods. The region surrounding the palpebral fissure was cropped. The operator marked the medial and lateral canthi on the image. To generate a palpebral fissure region of interest (ROI) using an interactive polygonal drawing method, the operator would first click on the medial or lateral canthus, then drag the mouse over the eyelid contour, and then click on the opposite canthus. the intercanthal distance and upper eyelid margin length were calculated by the MATLAB software. The picture was then binarized, and finally a circle was drawn around the eye with the line connecting the medial and lateral canthi serving as the diameter. (Fig. 1) Marking of images was done by only one operator. The operator marked the images two times in random order and the mean value of two measurements was used for the study. To address the issue of possible variability, we analyzed the agreement of the two measurements by calculating intraclass correlation coefficient (ICC) values.

**Fig. 2:**
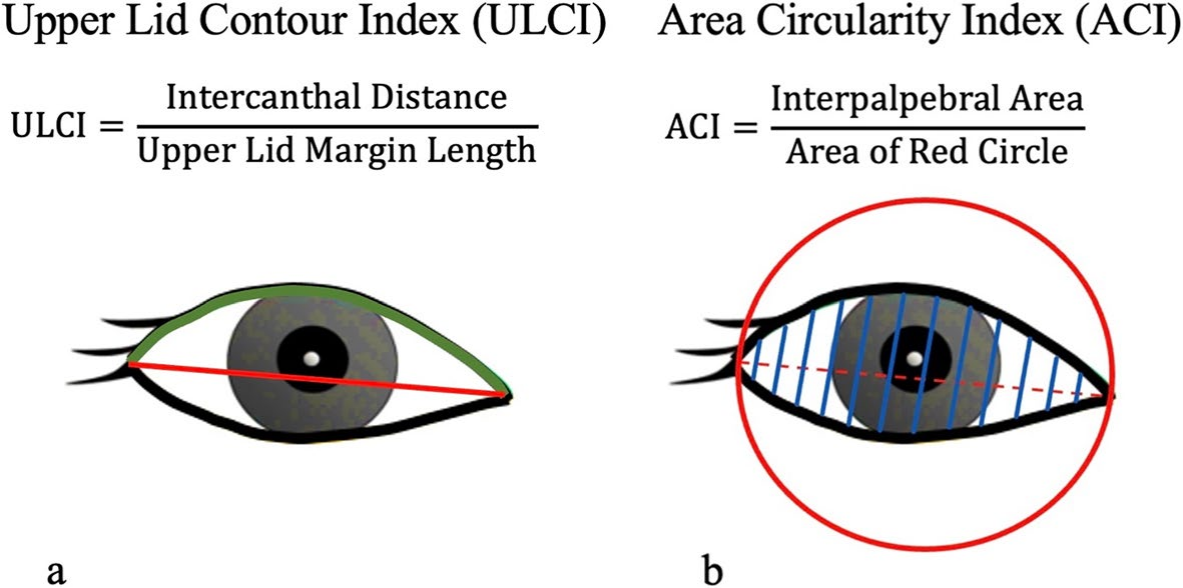
The formulas for calculating the three evaluated indices. The upper lid contour index (ULCI) was computed by dividing the intercanthal distance by the upper lid margin length (**a**). To create area circularity index (ACI), a circle was drawn around the eye, with the line connecting the medial and lateral canthi serving as the diameter. The ACI was calculated by dividing the interpalpebral area by the size of the circle (**b**)

Upper lid contour index (ULCI) was calculated by dividing the intercanthal distance by upper eyelid margin length (Fig. 2a). The area circularity index (ACI) is the second assessed index. To generate ACI, a circle was drawn around the eye, with the line between the medial and lateral canthi serving as the diameter. ACI was determined by dividing the interpalpebral area by the circle's area (Fig. 2b). The mean values of the indices described were compared between ptosis (mild, moderate, severe) and control groups. Also, the ratio (ULCI-ratio, ACI-ratio) of the indices between the study eye and the fellow eye was determined for each patient and compared across study groups.

### Statistical analysis

Statistical analysis was performed using SPSS (IBM Corp. Released 2017. IBM SPSS Statistics for Windows, Version 25.0. Armonk, NY: IBM Corp.). All results are expressed as means ± standard deviations (SD). Study groups were compared using a generalized estimating equation (GEE) analysis with a mixed model employed to account for the probable correlation between the two eyes. Adjustment for age and sex was performed. The Sidak correction was used for multiple comparisons. To evaluate the discrimination power of the parameters to detect mild from normal, moderate from mild and severe from moderate, Roc Curve analysis was performed. Youden's J statistic was used to identify the best cutoff values. A probability of *p* < 0.05 was considered statistically significant.

## Results

In the current investigation, a total of 106 eyes from 106 individuals were included. There were 25, 27, and 24 eyes in the mild, moderate, and severe ptosis groups, respectively, and 30 eyes in the control group with no ptosis. The demographics of the patients and the mean MRD-1 values of the enrolled and fellow eyes of the participants are presented in Table 1. The average age of patients in the control, mild, moderate, and severe ptosis groups was 45.27, 33.20, 34.04, and 35.46 years old, respectively. Mean MRD1 was 4.1 in the control group, 3.2 in the mild group, 2.2 in the moderate group, and 0.4 in the severe group. The MRD1 levels varied significantly across all groups. There was no statistically significant difference between the MRD1 values of the fellow eyes.

**Table 1:** The demographic data and MRD1 values for the study population

		**Group**				***P—value***
		**Normal**	**Mild**	**Moderate**	**Severe**	
**Sex**	Male	8 (26.7%)	8 (32.0%)	8 (29.6%)	11 (45.8%)	0.479
	Female	22 (73.3%)	17 (68.0%)	19 (70.4%)	13 (54.2%)	
**Laterality**	Right	13 (43.3%)	11 (44.0%)	12 (44.4%)	12 (50.0%)	0.963
	Left	17 (56.7%)	14 (56.0%)	15 (55.6%)	12 (50.0%)	
**Age**	Mean ± SD	45 ± 10	33 ± 15	34 ± 11	35 ± 21	** < 0.001**
	Range	25 to 67	3 to 67	16 to 57	4 to 67	
**MRD1**	Mean ± SD	4.1 ± 0.5	3.2 ± 0.6	2.2 ± 0.5	0.4 ± 1.1	** < 0.001**
	Range	3.3 to 5	2.1 to 4.5	1.5 to 3.5	-2 to 2.2	
**MRD1-Fellow eye**	Mean ± SD	4.2 ± 0.6	4.1 ± 0.7	4.1 ± 0.7	4.3 ± 1	0.454
	Range	2.8 to 5.5	2.8 to 5.5	2.8 to 5.4	1.8 to 6	

*MRD1* Margin reflex distance 1, *SD* standard deviation. Bold values are statistically significant

Two indices were evaluated in this study. Table 2 demonstrates the mean and inter-eye ratio values for ULCI and ACI in each of the four study groups. ULCI and ACI showed a statistically significant difference across groups (*p* < 0.001 for both). Furthermore, inter-eye ULCI-ratio and ACI-ratio had a statistically significant difference between groups (*p* = 0.002 and *p* < 0.001, respectively).

**Table 2:** The values of the mean, and the inter-eye ratio of the evaluated indices for the study groups

	**Groups**								***p-value†***
	**Normal**		**Mild**		**Moderate**		**Severe**		
	Mean ± SD	Range	Mean ± SD	Range	Mean ± SD	Range	Mean ± SD	Range	
**ULCI**	0.8253 ± 0.0218	0.7744 to 0.8699	0.8231 ± 0.0486	0.606 to 0.8597	0.8549 ± 0.032	0.7134 to 0.8919	0.8647 ± 0.049	0.6929 to 0.9207	** < 0.001**
**ACI**	0.3741 ± 0.0391	0.2766 to 0.468	0.3574 ± 0.0337	0.2983 to 0.426	0.3307 ± 0.0342	0.276 to 0.4329	0.2978 ± 0.0486	0.1866 to 0.3859	** < 0.001**
**ULCI-ratio**	0.9954 ± 0.0181	0.9369 to 1.0377	1.0116 ± 0.0856	0.7105 to 1.1951	1.0273 ± 0.049	0.8592 to 1.0889	1.0601 ± 0.0673	0.8386 to 1.1698	**0.002**
**ACI-ratio**	0.9812 ± 0.0473	0.8826 to 1.0723	0.9188 ± 0.0736	0.7285 to 1.0283	0.857 ± 0.0769	0.7158 to 1.0096	0.7417 ± 0.1089	0.4058 to 0.9875	** < 0.001**

*ULCI* upper lid contour index, *ACI* area circularity index, *SD*, standard deviation. Bold values are statistically significant

^†^ Based on GEE analysis, adjusted for age and sex

Pairwise comparison between groups was conducted (Table 3). ULCI of the control group had a statistically significant difference with moderate (*p* < 0.001) and severe groups (*p* = 0.003). Similarly, ULCI of the mild ptosis group had a statistically significant difference with the moderate (*p* = 0.004) and severe groups (*p* = 0.003). ACI of normal and mild, moderate, and severe ptosis groups were significantly different in all pairwise comparisons. (*p* < 0.05 for all).

**Table 3:** The pairwise comparison between groups for the evaluated indices

The pairwise comparison between groups for ULCI				
	Normal	Mild Ptosis	Moderate Ptosis	Severe Ptosis
Normal		*P* > 0.05	***P* < 0.001**	***P* = 0.003**
Mild Ptosis			***P* = 0.004**	***P* = 0.003**
Moderate Ptosis				P > 0.05
Severe Ptosis				
The pairwise comparison between groups for ULCI ratio				
	Normal	Mild Ptosis	Moderate Ptosis	Severe Ptosis
Normal		*P* > 0.05	*P* > 0.05	***P* < 0.001**
Mild Ptosis			*P* > 0.05	***P* = 0.024**
Moderate Ptosis				*P* > 0.05
Severe Ptosis				
The pairwise comparison between groups for ACI				
	Normal	Mild Ptosis	Moderate Ptosis	Severe Ptosis
Normal		***P* = 0.027**	***P* < 0.001**	***P* < 0.001**
Mild Ptosis			***P* = 0.003**	***P* < 0.001**
Moderate Ptosis				***P* = 0.009**
Severe Ptosis				
The pairwise comparison between groups for ACI Ratio				
	Normal	Mild Ptosis	Moderate Ptosis	Severe Ptosis
Normal		***P* = 0.001**	***P* < 0.001**	***P* < 0.001**
Mild Ptosis			***P* = 0.002**	***P* < 0.001**
Moderate Ptosis				***P* < 0.001**
Severe Ptosis				

*ULCI* upper lid contour index, *ACI* area circularity index. Bold values are statistically significant

*P*-values are adjusted for age and sex, based on Sidak’s method for multiple comparisons in GEE analysis

**Fig. 3:**
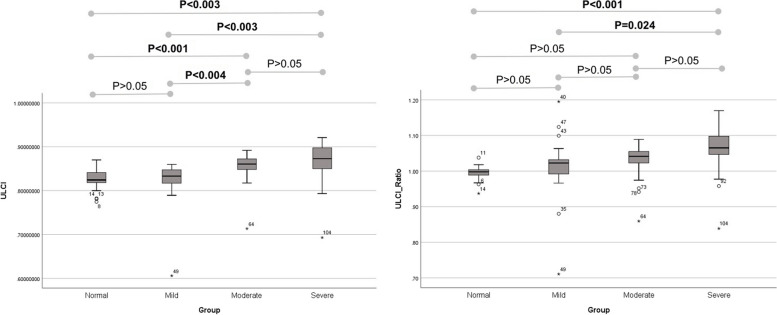
The pairwise comparison between groups for the evaluated indices: upper lid contour index (ULCI) and upper lid contour index-ratio (ULCI-ratio)

**Fig. 4:**
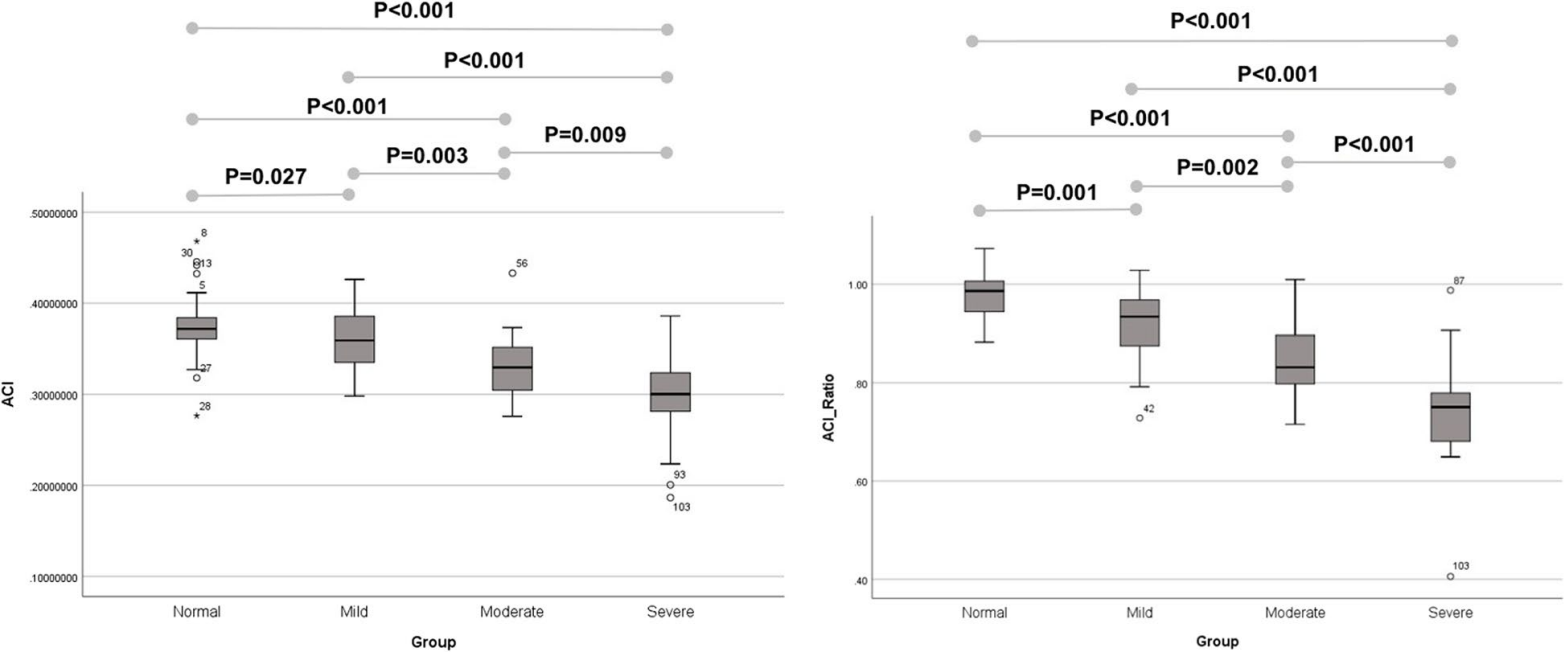
The pairwise comparison between groups for the evaluated indices: area circularity index (ACI) and area circularity index-ratio (ACI-ratio)

Regarding ULCI-ratio, the control and mild groups showed a significant difference from the severe group (*p* = 0.001 and *p* = 0.024, respectively), while other pairwise comparisons were not significantly different. (*p* > 0.05 for both) Interestingly, all pairwise intergroup comparisons were statistically significant for ACI-ratio. (Fig. 3, 4).

Analysis of the agreement of two measurements by the same operator by calculating intraclass correlation coefficient (ICC) values were as follows: Mild: 0.937, moderate: 0.940, and severe: 0.968 for ACI and mild: 0.908, moderate: 0.943, and severe: 0.894 for ULCI. There was a high agreement between the values.

## Discussion

Measuring lid curvature in an objective and reliable manner has been a long-desired goal for ophthalmic plastic surgeons [21]. Outcomes of upper eyelid surgeries typically have been reported qualitatively using descriptors such as "good" or "poor" [22]. MRD1 measurements, whether manual or on digital photographs, are an important part of the assessment of upper lid position and surgery results. However, their reliability is limited. It has been found that physicians' assessed MRD1 might vary by up to 0.5 mm on average [7]. Furthermore, eyelid abnormalities are not confined to a single central spot where MRD1 is assessed. Therefore, more effective approaches are required to characterize the eyelid shapes.

In the current study, we evaluated two novel indices and their effectiveness in discriminating different severities of ptosis and a group of control patients. Each index was compared between groups in two formats: the mean, and the inter-eye ratio values.

While there was a significant difference present between the groups, pairwise comparisons revealed that ULCI was not significantly different between control and mild and also moderate and severe groups. ULCI-ratio also couldn't distinguish between the control and mild ptosis groups. The ACI divides the area of the interpalpebral fissure by the area of the circle whose diameter is the intercanthal line. Mean ACI was significantly different between mild, moderate, and severe ptosis and control group. Similarly, there was a statistically significant difference in ACI-ratio between all four study groups even in pairwise comparisons.

Any disease of the upper eyelids affects the eyelid curvature nonuniformly, changing the height and curvature of some points more than others. ULCI, due to its formula, generates a general index of the whole eyelid status. Although this might be useful in describing the general eyelid shape, it will inevitably lessen the impact of any deformities. Hence, ULCI failed to distinguish between all of the four study groups. ACI can have the same tendency of generalizing eyelid morphology, but it successfully distinguished control and different severities of ptosis from each other. This finding suggests that indices based on area measures and describing eyelid circularity might have good sensitivity for describing deformities.

Other groups have proposed methods to quantify eyelid contour. Multiple radial mid-pupil lid distances (MPLD) [16] involve marking the center of the pupil and drawing multiple lines ranging from 0 to 180° at 15° degree intervals from the pupil center up to where the lines meet the upper eyelid margin. This system has been used to quantify contour in normal patients [23], thyroid eye disease [24], and blepharoptosis [10]. MPLDs provide robust information on curvature compared to MRD1, and temporal and nasal MPLD ratios can be calculated to assess asymmetries in the eyelid shape. While giving a comprehensive outline of the contour, when used for comparing pre and postoperative curvature or patients with different eyelid heights, MPLD lines intersect the eyelid at points that don't correspond to each other, which might introduce some errors to the assessment. Also, applying MPLD to severe ptosis patients with obstructed pupil centers can be challenging [17].

Danesh et al. have employed multiple lines parallel to the MRD1. The lines are spaced in 2 mm intervals medial and lateral to the central MRD1. They evaluated Müller’s muscle-conjunctival resection and external levator resection for blepharoptosis and reported a higher eyelid height for the external levator resection technique at the 2 and 4 mm temporal positions [17]. Equiterio et al. have proposed distributing the same type of lines at 10% intervals between the temporal corneal limbus and the lateral canthus [12]. In contrast to using a fixed distance between the lines, this method accounts for the variability of the eyelid length between different subjects. By using this method and calculating the ratio of the line 10% medial to the lateral canthus to MRD1, they could objectively predict the presence of lateral lid flare. This method currently has not been used for evaluating other conditions.

Bezier curves have been studied extensively to fit a line based on polynomial functions to the eyelid contour. Their usage is limited by the complexity of line fittings and pathologic conditions of the upper eyelid not adhering strictly to a polynomial function [13, 14]. Deep learning methods have been used to study eyelid curvature recently. By training a neural network, Lou et al. successfully segmented the eye to medial, corneal, and lateral areas and measured different indices such as MRD1, upper and lower lid length, and MPLD with great accuracy and reproducibility [15].

We also evaluated the correlation between ULCI (*r* = -0.403, *p* = 0.003), ULCI-ratio (*r* = -0.392, *p* = 0.004), ACI (*r* = 0.689, *p* ≤ 0.001), and ACI-ratio (*r* = 0.590, *p* ≤ 0.001) with MRD1. The results showed a mild to moderate correlation as would be expected as they are all quantifications of the same phenomenon namely ptosis. However, the correlations are far from perfect which indicates that while there is some agreement between them, they are not the same. This is a consequence of our indices reflecting a measure of the whole eyelid contour compared to just one point in MRD1.

In our study, ACI proved to be sensitive for distinguishing ptosis patients by incorporating the whole eyelid data and producing an index of the general status of the eyelids. The ACI-ratio, which was computed using the normal fellow eye of the patients, demonstrated a higher level of performance and was able to differentiate between all four groups of control, mild, moderate, and severe ptosis patients. This demonstrates the need of comparing the two eyes of unilaterally afflicted individuals to gain the most information on eyelid curvature. Incorporating data from both eyes in this manner may also reduce the effect of imaging circumstances that affect both eyes roughly equally. For instance, a vertical tilt in the patient's head or changes in the imaging device's plane would likely result in a higher variance in the measurement in one eye compared to an inter-eye ratio assessment. Assessing eyelid indices in the ratio format and binarizing digital photographs helps reduce variables that might effect eyelid digital photography, such as camera distance from the patient and ambient lighting.

This study has some limitations. The marking of the canthi and palpebral length are operator dependent. A fully automatic solution would be desirable. Digital photographs were taken from subjects during a routine clinic visit, and the variability of successful measurements should be evaluated prior to the broader employment of this method. One of the limitations of our study is that we were unable to control for the distribution of sex and age in each group due to the retrospective nature of this investigation. But, given the variations in palpebral fissure anatomy observed among individuals of different age, sex, and ethnicity, we have put forth the concept of indices in the form of ratios (ULCI-ratio, ACI-ratio). These ratios serve to compare each eye of a patient with its corresponding fellow eye. The utilization of ratios as an indicator for comparing diverse anatomical characteristics in eyes across varying age groups, genders, and ethnicities has the potential to mitigate the influence of inherent disparities. Another limitation of our study was that the population in our study exclusively comprised individuals of Persian Middle-Eastern Descent. Thus, it should be noted that the findings of our study may not be generalizable to other ethnicities. Also, standardized images may not always be available in clinical practice. Another limitation of our study was that we did not investigate the relationship of these new indices with palpebral fissure size and levator function. Lastly, the study sample size was limited, and larger sample sizes are required to confirm these results.

## Conclusion

We proposed two new indices and evaluated their effectiveness for distinguishing between control and ptosis patients. ACI, describing the circularity of the eyelids using area measures, could distinguish control group and different ptosis severities. ACI-ratio had greater power by including data from normal fellow eyes. Further studies are required to confirm our results and evaluate the effectiveness of ACI in describing other eyelid pathologies.

## Data Availability

The datasets used and/or analysed during the current study are available from the corresponding author on reasonable request.
